# Unlocking Security for Comprehensive Electroencephalogram-Based User Authentication Systems

**DOI:** 10.3390/s24247919

**Published:** 2024-12-11

**Authors:** Adnan Elahi Khan Khalil, Jesus Arturo Perez-Diaz, Jose Antonio Cantoral-Ceballos, Javier M. Antelis

**Affiliations:** School of Engineering and Sciences, Tecnologico de Monterrey, Monterrey 64700, Nuevo Leon, Mexico; a00836552@tec.mx (A.E.K.K.); joseantonio.cantoral@tec.mx (J.A.C.-C.); mauricio.antelis@tec.mx (J.M.A.)

**Keywords:** multi-factor authentication, user authentication, user identification, electroencephalogram (EEG), MLP neural networks, machine learning, P300 potentials

## Abstract

With recent significant advancements in artificial intelligence, the necessity for more reliable recognition systems has rapidly increased to safeguard individual assets. The use of brain signals for authentication has gained substantial interest within the scientific community over the past decade. Most previous efforts have focused on identifying distinctive information within electroencephalogram (EEG) recordings. In this study, an EEG-based user authentication scheme is presented, employing a multi-layer perceptron feedforward neural network (MLP FFNN). The scheme utilizes P300 potentials derived from EEG signals, focusing on the user’s intent to select specific characters. This approach involves two phases: user identification and user authentication. Both phases utilize EEG recordings of brain signals, data preprocessing, a database to store and manage these recordings for efficient retrieval and organization, and feature extraction using mutual information (MI) from selected EEG data segments, specifically targeting power spectral density (PSD) across five frequency bands. The user identification phase employs multi-class classifiers to predict the identity of a user from a set of enrolled users. The user authentication phase associates the predicted user identities with user labels using probability assessments, verifying the claimed identity as either genuine or an impostor. This scheme combines EEG data segments with user mapping, confidence calculations, and claimed user verification for robust authentication. It also accommodates new users by transforming EEG data into feature vectors without the need for retraining. The model extracts selected features to identify users and to classify the input based on these features to authenticate the user. The experiments show that the proposed scheme can achieve 97% accuracy in EEG-based user identification and authentication.

## 1. Introduction

The threat of personal data exposure through unauthorized access has never been as imminent as it is in recent times [1]. Systems need different types of authentication to increase security, and traditional forms of authentication, such as PINs and passwords, tend to be vulnerable to forgery or easily stolen. Due to the growing number of security vulnerabilities, the need for better and safer authentication has gained more importance than ever before. Authentication is a means of verifying an identity that is claimed by utilizing different types of assets or data [2]. These authentication techniques utilize an array of user attributes, including possession (something the individual owns, e.g., a digital certificate, smart card, portable device, etc.), knowledge (something the individual knows, such as a password), or inherent traits (something the individual has, i.e., biometrics such as fingerprints, facial, or voice recognition). Biometric features, which are characterized by “something the user is”, play a very important role in modern authentication systems. Commercially available biometric features include physical aspects such as fingerprints, facial characteristics, iris scans, and palm vein patterns, as well as behavioral aspects like keystroke patterns, signature analysis, and voice recognition [3]. In this context, brain signals, classified as a subset of behavioral features, have gained interest due to their distinct advantages, particularly their concealed yet dependable nature [4]. Brain activities are unique in the way they closely correlate with an individual´s mental state, actions, and cognitive conditions [5]. These signals unveil both psychological and behavioral characteristics. The remarkable attributes of brain activities are their state-dependent dynamic behavior and their distinctive resistance to spoofing attacks, including attempts to imitate or forge a user [6]. Brain activity can be measured by an “electroencephalogram” (EEG), a technology involving the placement of electrodes on the scalp to record electrical signals [7]. These signals are produced by the synaptic activation of a group of neurons. In recent years, EEG signal analysis has gained attention because of its informative content, and it has played a pivotal role in several research fields, such as medicine, biometrics, and many other research fields [8]. By capitalizing on the distinct advantages of brain signals, [9] enhanced security and privacy measures in biometric authentication systems. EEG-based user authentication aligns with the principles of EEG-based biometrics, utilizing the unique nature of brain signals to verify the identity of users [10]. By analyzing the distinctive EEG patterns, it ensures that only authorized users can access the system. Moreover, EEG-based user authentication contributes to improving user privacy, thereby introducing an extra layer of confidentiality [11], addressing security concerns while maintaining user-friendly and scalable authentication procedures that manage growing user bases without compromising performance and security. Furthermore, EEG-based user authentication, which relies on the behavioral and psychological traits of users, offers robust protection against spoofing attacks and attempts to impersonate individuals [12]. These attributes make it a promising and secure option for modern authentication systems [13]. EEG-based user authentication not only combines the unique attributes of brain activity but also aligns with the evolving landscape of biometric authentication for enhanced security and user privacy. This research seeks to address significant challenges in the field of EEG-based user authentication. These challenges include comprehensiveness, user-friendliness, and the preservation of user confidentiality in real-time EEG-based authentication scenarios. In response to these challenges, it also introduces an innovative and comprehensive EEG-based user authentication scheme based on deep neural network techniques. The key contributions of the proposed study are as follows:

1. A unique approach for user identification and authentication by integrating selected features obtained through mutual information analysis. Specifically, it highlights the utilization of power spectral density (PSD) values, enhancing its ability to extract relevant and discriminative features, making it more significant for user recognition purposes compared to conventional methods.
2. A comprehensive architecture for EEG-based user identification and authentication, enabling the verification of newly enrolled users even without their EEG data during the initial training phase. This adaptability allows the system to effectively scale and accommodate a growing user base.
3. An approach that emphasizes utilizing selected features instead of directly storing or processing raw EEG data, significantly enhancing privacy by eliminating the need to retain sensitive raw EEG data that contain crucial information essential for analysis and interpretation.
4. A novel approach for EEG-based user authentication, which involves the calculation of predicted labels from classified EEG data segments. The final decision depends on the probability of the majority predicted label, with a predefined threshold used to ensure the authentication of genuine users and imposters.

The rest of this paper is organized as follows. Section 2 discusses the challenges in the field of EEG-based user authentication and their implications in detail. Section 2 also illustrates the current and previous common EEG-based authentication techniques using various machine learning and deep learning models. The problem statement for EEG-based user identification and authentication tasks, the architecture utilized in this research, and the representation of EEG data segments used as input, along with preprocessing and feature extraction using the mutual information of selected features, are discussed and explained in Section 3. Furthermore, the core of the proposed user authentication scheme is detailed in the subsection on probability assessment, while the verification process is described in the subsection on the validation procedure. The tests, experiments, and results are analyzed and presented in Section 4, with an emphasis on user identification and authentication in particular. Section 5 concludes with a comparative discussion highlighting the novelty of the proposed scheme in relation to state-of-the-art works, and the conclusions are drawn in Section 6.

## 2. Related Works

EEG-based user authentication presents several significant challenges that must be addressed for practical and real-time applications. One main challenge is designing a procedure that effectively identifies and authenticates users through their EEG patterns [14]. Current systems employ a variety of techniques, as explored in [15], but they often face issues like overfitting due to the high number of learnable parameters relative to the available training data [16,17]. Although the effectiveness of these models has increased, overfitting remains a frequent problem since there are many more learnable parameters than there are available training data. In general, EEG-based user authentication is considered a classification problem, in which EEG signals from a group of users are utilized to train a classification model. During the EEG recording, the signals are subjected to preprocessing, and then a set of distinctive features is extracted [18]. Subsequently, a classifier is trained using these extracted features, which is employed to perform two main functions: user identification and user authentication. In user identification, the classifier is used to determine the identity from a given set of EEG records, while in user authentication, the classifier verifies whether two EEG records belong to the same user. In this study, the primary focus is on EEG-based user authentication, incorporating both user identification and verification. It is important to emphasize that for a classifier to work effectively, the user needs to replicate the same task they performed during the initial recording.

Generally, EEG-based biometric systems rely on shallow classifiers, like linear discriminant analysis [19], support vector machine [20], etc. However, in recent years, deep learning models [21] have shown promising results in terms of accuracy and enhanced authentication. Although these classifiers have achieved better accuracy in EEG-based biometric applications, they still face a critical challenge known as “comprehensiveness”. This challenge refers to the requirement that the authentication factor should be applicable to every individual, not just those included in the training dataset [22]. Current EEG-based authentication systems are limited, as they can only identify or authenticate users included in the initial training dataset. When a new user needs to register, their EEG data must be added, and the classifier retrained. This approach is impractical for real-time applications and suits systems with a predefined, limited user set. However, unlike traditional models, neural networks can accommodate new data without retraining from scratch. Few studies have explored techniques in EEG-based recognition that allow the inclusion of new users without requiring complete retraining of the model. In [23], the author proposed a transfer learning technique to adapt an existing model for new users, but it struggled with accommodating large sets of new users. An EEG feature extractor model proposed in [24] used deep learning for feature extraction, but instead of using extracted feature vectors directly, it employed them in a fully connected neural network, making it similar to a classification model. Addressing this challenge of scalability is crucial for practical, real-time EEG-based user authentication.

Another significant challenge of EEG-based user authentication is the number of electrodes required. The authors of [25] used two electrodes for user authentication. To achieve better accuracy with fewer electrodes, the authors of [26] proposed a user authentication method. In [27], the number of electrodes employed was 33, significantly more than the number commonly found in most commercial EEG headsets. Some methods assume EEG data are sampled with up to 64 channels [28], which is typically found in advanced medical devices. However, using many electrodes presents challenges in data acquisition and reduces user satisfaction. For practical use, a user authentication system should rely on a limited number of electrodes. The most important challenge in the methods proposed is the lack of attention to user privacy [29]. EEG-based user authentication typically compares two EEG samples to verify whether they belong to the same person. However, storing EEG data for each user can expose sensitive information, such as gender, age, medical status, and other data [30]. Methods should adopt a password-based approach, where the server stores a value instead of the actual data, preserving privacy while verifying the identity. Currently, only a small fraction of techniques offer such privacy-preserving capabilities. In this research study, the main focus is on addressing various challenges related to EEG-based user authentication by employing an FFNN to accurately authenticate users [31] through the analysis of EEG signals. The aim of this study is to enhance the practical applicability of user authentication systems by proposing a method that maintains high accuracy and efficiency compared to state-of-the-art approaches. Furthermore, it is expected to extend the system’s usability to individuals who are not part of the training database by utilizing a multi-class classification technique to distinguish users based on their unique EEG patterns, making comprehensive EEG-based user authentication more achievable while preserving user privacy.

Developing an effective EEG-based recognition technique starts with extracting relevant features from raw EEG data. Since EEG signals are complex and unstructured, it is crucial to extract distinctive features before training machine learning models. The choice of feature extraction methods significantly affects the accuracy of the user identification and authentication system. Several well-established techniques are employed for EEG feature extraction, including the autoregressive model [32] for capturing temporal dependencies, power spectral density [33] for frequency-based analysis, wavelet transform [34] for transient and frequency-specific information, principal component analysis [35] for reducing the complexity of EEG data while retaining relevant information, time-domain features [36] for statistical information about the EEG signals, and the Fourier transform [37] for analyzing signals in the frequency domain. Once these distinguishing features are extracted from the EEG data, the user identification and authentication processes can typically advance through different approaches.

The “feature-based approach” involves extracting relevant features from EEG data. For example, Alsumari et al. [38] designed an EEG-based recognition system based on a one-dimensional temporal domain representation of EEG data. Wilaiprasitporn et al. [39] proposed an EEG-based biometric system for identifying users by extracting 15 statistical features using neighborhood component analysis. Białas et al. [40] proposed an EEG-based authentication system combining signal analysis and user image verification, integrating a random forest (RF) classifier with a mobile app, achieving 83% classification accuracy. With respect to the algorithms used for EEG-based user identification and authentication, the most commonly used one is SVM. Leon et al. [41] enhanced the validation of BCI using SSVEP for multi-class classification. Schons et al. [42] developed an EEG-based biometric system using a CNN with 64-channel inputs and data augmentation, achieving 99% accuracy for 109 users. However, it relies on 12-second EEG segments, which is not ideal for real-time applications. An innovative EEG authentication framework was introduced by Bingkun et al. [43] for end-to-end user verification using a CNN on two public datasets, demonstrating promising results. However, the small sample size for channel selection and the lack of evaluation factors pose challenges to the scalability of the system. Debie et al. [44] used an EEG fusion method with a CNN to improve user authentication, demonstrating better performance and reliability under cross-sectional conditions compared to single-protocol models. Maiorana et al. [45] used hidden Markov models for EEG-based user recognition, spanning six trials and two sessions per subject over three years. While the study addressed longevity factors, it lacked detailed feature selection criteria and did not explore EEG signal stability beyond this period. Kim et al. [46] used local function networks (FNs) for EEG-based user authentication, focusing on quadrantal FNs under different resting conditions. While showing promising results, their approach also used t-SNE for visualization, which has limitations such as sensitivity to hyperparameters, poor scalability, and interpretability problems. The feature extraction method plays a vital role in the effectiveness of EEG-based user authentication. For example, using machine learning models and methods for capturing meaningful information from the EEG signals to identify and authenticate users accurately is essential.

MLP neural networks [47] are ideal for EEG-based user authentication due to their ability to automatically identify relevant features from raw EEG data, eliminating the need for manual feature extraction and simplifying the authentication process. The built-in feature extraction capability simplifies the whole authentication process, increasing its effectiveness and reducing the complexity of system design. MLP neural networks have achieved notable success in EEG-based user authentication. Waili et al. [48] proposed an MLP network using Daubechies (d8) wavelet for feature extraction, achieving 73.65% accuracy in biometric recognition. However, the choice of filter parameters and their impact on EEG signal quality were not discussed. Haukipuro et al. [49] used a single-electrode EEG device for mobile authentication, performing multi-step processing with 27 users in a real-life scenario, achieving 85.30% accuracy. However, the authentication error increased when new users were added, indicating potential limitations in the feature space or user overlap. Using auditory stimuli at rest for EEG-based authentication was presented in [50], with three classifiers utilized (MLP, KNN, and XGBoost), achieving an accuracy of 69% based on the auditory stimulus. Despite the low accuracy, the study lacked details on the sample size, user demographics, and auditory stimuli used for authentication. Liew et al. [51] proposed an incremental fuzzy-rough nearest neighborhood (IncFRNN) technique for biometric authentication using visual evoked potentials from 37 users, achieving 87% accuracy. However, the validation process to reduce overfitting was not clearly explained. EEG-based biometric authentication was performed using eye-blinking signals [52], with stationary wavelet transforms and independent component analysis (SWT+ICA) for preprocessing. An artificial neural network (ANN) achieved 81% accuracy, but the study omitted details on the feature extraction and fusion techniques used. The reviewed papers listed in Table 1 focused mostly on EEG-based user identification rather than user authentication. Although several machine learning and deep learning models have been employed for EEG-based authentication, integrating multi-factor authentication (MFA) methods could further enhance the security and robustness of these systems. For example, Theodoropoulos et al. [53] and P.D. et al. [54] demonstrated how integrating OTPs and cryptographic techniques with multi-factor frameworks can strengthen authentication mechanisms in various applications. Similarly, Cheng et al. [55] showcased the effectiveness of hybrid EEG and eye movement-based authentication systems, achieving superior accuracy and reliability compared to single-modality approaches, which highlights the potential of combining physiological and cryptographic factors for more secure hybrid EEG-based authentication systems.

The proposed user authentication approach is a promising method that focuses on power spectral density (PSD) for extracting features from five frequency bands: alpha, beta, theta, gamma, and delta. This feature extraction utilizes mutual information (MI) and employs a feedforward neural network (FFNN) for classification. Notably, the use of the P300 speller paradigm with a reduced number of channels (only eight channels) distinguishes this approach from others. The proposed method not only achieves improved accuracy but also simplifies EEG-based user identification and authentication by performing a probability assessment based on predicted labels to accurately differentiate between genuine users and impostors.

## 3. Methodology

This section discusses the methodology employed for the proposed EEG-based user identification and authentication scheme. The primary objective of this research is to recognize users based on their EEG signals using a machine learning model. It begins by describing the identification and authentication tasks, followed by an explanation of feature extraction using power spectral density (PSD) and feature selection based on mutual information (MI). Next, the technical details of the classification models used in this study, with an emphasis on the FFNN, are presented. Finally, the validation procedure using a publicly available dataset and the performance metrics are described in detail.

### 3.1. EEG-Based User Identification and Authentication Tasks

#### 3.1.1. Problem Statement

Let $R^{M\times T}$ denote the space of EEG signal segments taken from N different users. Each EEG signal segment, denoted as $X={\left[x_{1},x_{2},\cdots ,x_{M}\right]}^{⊤}$, consists of *M* channels sampled at *T* time points, where $x_{i}={\left[x_{i}\left(0\right);x_{i}\left(1\right);\cdots ;x_{i}\left(T-1\right)\right]}^{⊤}$ is the activity of the *i*-th channel.

Furthermore, let y∈{1,2,…,N} be the set of labels for N users. There are two EEG-based user recognition tasks: a user identification task and a user authentication task.

#### 3.1.2. EEG-Based User Identification Task

**Figure 1:**
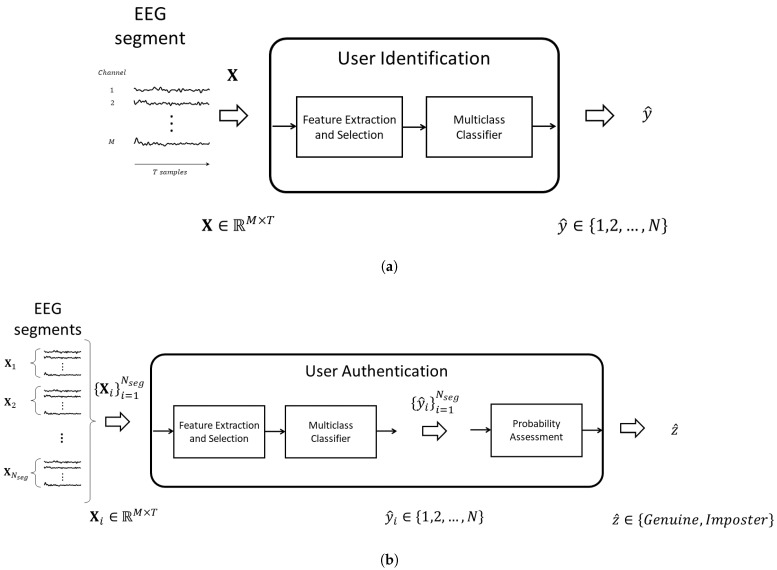
This figure shows two EEG-based tasks: (**a**) user identification and (**b**) user authentication. The identification task classifies which user the EEG segment belongs to, while the authentication task validates whether the user is genuine or an imposter based on the EEG signals.

In the EEG-based user identification task, the objective is to determine which specific user from a set of known users an input EEG signal segment belongs to, i.e., predict the user label $\hat{y}\in \left\{1,2,\ldots ,N\right\}$ from an EEG data segment $X\in R^{M\times T}$. Figure 1a illustrates the process of how EEG-based user identification is performed. This recognition task is based on a machine learning model that first extracts and selects features and then uses a classification model to predict the user label.

#### 3.1.3. EEG-Based User Authentication Task

The aim of the EEG-based user authentication task is to validate the claimed identity of a user as either genuine or an imposter based on multiple EEG signal records, i.e., assign an authentication label record, to estimate an authentication label $\hat{z}\in \left\{\mathrm{genuine},\mathrm{imposter}\right\}$ from a set of several EEG data segments ${\left\{X_{i}\right\}}_{i=1}^{N_{seg}}$, where $X_{i}\in R^{M\times T}$ and $N_{seg}$ is the total number of segments. A description of the EEG-based user authentication task is presented in Figure 1b. This recognition task first estimates the user identification label for each EEG data segment based on the machine learning model and then uses the set of estimated labels, along with the probability assessment, to determine the identity as either a genuine user or an imposter.

### 3.2. Feature Extraction and Feature Selection

#### 3.2.1. Power Spectral Density (PSD)-Based Features

In this study, the frequency domain features characterizing brain rhythms are computed from the EEG data segments. The power spectral density (PSD) values are calculated using a modified periodogram [56]. Given the signal of an EEG channel x=[x(0), ${x\left(1\right),\cdots ,x\left(T-1\right)]}^{⊤}$, the PSD is calculated with the fast Fourier transform (FFT) using the following equation:

(1) $$\mathrm{PSD}\left(f\right)=\frac{1}{N_{x}}\left|\mathrm{FFT}{\left[x_{\mathrm{windowed}}\right]}^{2}\right|$$

where PSD(f) is the PSD at frequency *f*, $x_{\mathrm{windowed}}$ is the windowed version of the signal *x*, and $N_{x}$ is the number of EEG data samples.

After calculating the PSD, the power values are summed within the predefined frequency bands: delta (1–4 Hz), theta (4–8 Hz), alpha (8–12 Hz), beta (12–30 Hz), and gamma (30–50 Hz). In total, five power spectral values are computed for each EEG channel. Therefore, the resulting feature vector is $x\in R^{P\times 1}$, where *P* represents the total number of features derived from the EEG signals. This is defined as the product of *M* channels (the number of EEG recording channels) and the five frequency band spectral values (the spectral values calculated for each frequency band).

#### 3.2.2. Feature Selection Using Mutual Information

The goal of the feature selection method based on mutual information is to minimize redundancy among the selected features while maximizing the average mutual information between each feature vector. To achieve a low-dimensional representation of the original feature vector while maintaining high discriminative power, the mutual information (MI) method is employed for selecting features [57]. The MI method measures the dependency between two feature vectors. It is a measure of how similar two feature vectors are to one another; a high mutual information value denotes similarity and a significant decrease in uncertainty, whereas a low mutual information value shows dissimilarity and a small reduction in uncertainty. The MI of two feature vectors, $X_{i}$ and $Y_{i}$, whose joint probability distribution is defined as $P(x_{i},y_{i})$, is calculated as follows:

(2) $$\mathrm{MI}\left(X_{i},Y_{i}\right)=\sum P\left(x_{i},y_{i}\right)\log\left(\frac{P(x_{i},y_{i})}{P\left(x_{i}\right)P\left(y_{i}\right)}\right)$$

where $\mathrm{MI}(X_{i},Y_{i})$ is the mutual information of the *i*-th feature, $X_{i}$ represents the feature vector (input data), $y_{i}$ represents the user labels, $P(x_{i},y_{i})$ represents the joint probability of feature $x_{i}$ and user label $Y_{i}$, and $P(x_{i})$, $P(y_{i})$ are the marginal probability distributions of feature $x_{i}$ and user label $y_{i}$. The sum is taken over all possible values of x, y.

A feature selection method was implemented to select the best features based on their mutual information with the target labels. It computed the mutual information between each feature and the labels. Subsequently, the best *Q* features with the highest mutual information scores were selected, resulting in the feature vector described as follows:

(3) $$\begin{array}{cc} x_{i} & \in R^{P\times 1}\to x_{i}\in R^{Q\times 1} \end{array}$$

where Q<P.

### 3.3. Classification Models

For EEG-based user identification and authentication, the core component is the utilization of a classifier. A feedforward neural network (FFNN) is employed to enhance the efficiency of user authentication. Additionally, multiple classifiers are used for benchmarking. Other classifiers, such as support vector machine (SVM), k-nearest neighbors (KNN), random forest (RF), and XGBoost, are utilized for the training and evaluation of multi-class classification of EEG data.

#### 3.3.1. Multi-Layer Perceptron Feedforward Neural Network (MLP FFNN)

**Figure 2:**
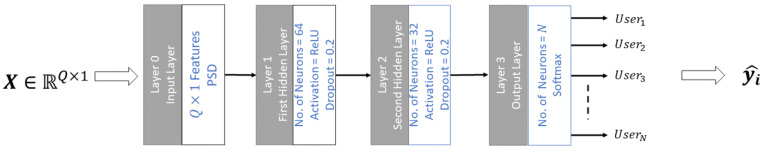
Architecture of the proposed MLP FFNN for user identification and authentication.

An FFNN [58] is a model employed for supervised learning that utilizes the backpropagation algorithm. It is preferred for its fast operation and ease of implementation. While FFNNs can have a large number of parameters depending on their depth and architecture, they are known for their flexibility and capability in handling non-linear relationships within EEG data and, therefore, generalize well on unseen data. The overall architecture of the proposed model is illustrated in Figure 2 and consists of three fully connected layers: the first hidden layer with 64 neurons, the second hidden layer with 32 neurons, and the output layer with neurons equivalent to the number of classes in the classification task. Rectified linear unit (ReLU) activation functions are employed after the first and second hidden layers for non-linearity. During the forward pass, input data move through the network, passing through each layer successively while applying the activation functions. Each hidden layer is followed by dropout regularization at a rate of 0.2, which randomly removes 20% of the neurons to reduce overfitting. Cross-entropy loss is utilized as the loss function, and the Adam optimizer is employed for parameter optimization. Therefore, this model reduces the risk of overfitting the data, especially when dealing with limited training data, thereby enhancing its ability to generalize better on unseen data. The output layer, which consists of *N* neurons (where *N* is the number of users), utilizes a softmax activation function to classify and identify users. The generalized representation of the model for user identification is as follows:

(4) $$\hat{y}=g\left(X_{j};\phi \right)$$

where $X_{j}$ represents the input feature vector with Q×1; ϕ represents the parameters of the model, including the weights, biases, and activation functions; and $\hat{y}$ is the predicted label for the input feature vector Q×1.

#### 3.3.2. Support Vector Machine (SVM)

Support vector machine (SVM) is a binary classifier that can be expanded into a multi-class classifier. It is selected due to its high generalization ability, and it has been proven to work well for classification and EEG-based identification and authentication [59]. Considering the model assigns a class label to the given input sample x, the decision function $f_{\mathrm{SVM}}\left(x\right)$ for user identification can be represented by

(5) $$f_{\mathrm{SVM}}\left(x\right)=\mathrm{sign}\left(\sum_{i=1}^{N}\gamma_{i}y_{i}\langle x_{i},x\rangle +b\right)$$

where $\gamma_{i}$ represents the Lagrange multipliers, $y_{i}$ represents the class labels, $x_{i}$ represents the support vectors, $\left(x_{i},x\right)$ represents the dot product between the support vectors and input sample x, *b* is the bias term, and *N* is the number of users. For identification, the decision function can be used to determine whether the given input sample x belongs to the specific class.

SVM was employed for the classification of EEG data in the proposed study while maintaining a balanced class distribution. The model calculates the accuracy of the model’s predictions and prints a classification report, which includes metrics such as precision, recall, F1 score, and accuracy for each user.

#### 3.3.3. K-Nearest Neighbors (KNN)

KNN has shown better results in EEG signal classification compared to other classification algorithms such as SVM, RF, and XGB [60]. This classification method is based on a user-defined constant integer k, which determines the class assignment of a new data point. In this study, unseen input data (e.g., EEG features) are given, and KNN calculates the distances between the unseen data and all data in the testing dataset. It then selects the k-nearest neighbors based on these distances, where k is a predefined hyperparameter. The algorithm assigns the class label that is most frequent among the k-nearest neighbors to the unseen input sample. This assigned class label represents the identified user. However, instead of assigning the class label directly, the algorithm computes a confidence score based on the majority votes of each class among the k-nearest neighbors. If the confidence score exceeds a predefined threshold, the algorithm accepts the user as genuine. Otherwise, it rejects the user as an imposter.

#### 3.3.4. Random Forest (RF)

Random forest is a method that combines several individual decision tree classifiers [61], whose result is the most voted class. RF has a relatively fast training time and can be applied to regression or classification problems. Some of the advantages of RF are that it has a small number of user-defined parameters, is easy to use in high-dimensional problems since it does not need any adaptation, and is easy to implement. In decision tree classifiers, the leaves represent the labels, while the branches represent the decision paths based on feature values. Several decision trees are built in a random forest (RF), and the final classification is obtained by combining each tree’s individual predictions. In this sense, the model learns how to differentiate the labels depending on the value of the features, hence creating new branches from each leave. In this study, 100 decision tree classifier ensembles with a fixed number of seeds (random state = 123) were implemented to ensure reproducibility, allowing trees to grow to their full depth to capture complex patterns in the data. The hyperparameters were fine-tuned while adjusting the number of estimators and the random seeds. Adjusting the random seeds ensured that the randomness in the data sampling and tree-building processes was controlled. Grid search was employed to explore and optimize various hyperparameters, potentially improving the model’s accuracy and robustness.

#### 3.3.5. XGBoost (XGB)

XGBoost is used for supervised learning problems. It is a machine learning algorithm that provides a highly accurate implementation of a gradient-boosting framework [62]. It combines a set of weaker models to provide accurate predictions as follows:

(6) $$x_{j}=\sum_{m=1}^{M}f_{k}$$

where *m* is the number of decision trees and $f_{k}$ is the prediction from the decision tree.

In this study, the XGBoost classifier was configured with several key hyperparameters: 100 boosting trees with the maximum depth set to 2, which restricts each tree to a maximum depth of 2, thus controlling the model’s complexity and helping prevent overfitting. The learning rate was set to 0.01 to adjust the step size during optimization for better convergence of the loss function. The hyperparameters were tuned using grid search and cross-validation, and parameters such as the number of trees, depth, and learning rate were optimized to maximize accuracy and model robustness. A learning rate of 0.01 and 100 trees with a maximum depth of 2 was the best-performing configuration, which achieved an optimal balance between predictive performance and complexity.

### 3.4. Probability Assessment

After feature extraction and classification, the predicted labels for unseen EEG data segments are obtained as described previously. In this study, user authentication is carried out by utilizing these predicted labels. Given the set of the predicted labels ${\hat{y}}_{i}\;\mathrm{for}\;i=1\;\mathrm{to}\;N$, the frequency of occurrence for each label is calculated by solving:

(7) $$V\left(j\right)=\sum_{i=1}^{N_{\mathrm{seg}}}\delta \left({\hat{y}}_{i},j\right),\;\;\forall \;\;j=1,2,...,N$$

where V(j) is the number of times the *j*-th label is predicted and $\delta ({\hat{y}}_{i},j)$ is the Kronecker delta function, which is equal to 1 if ${\hat{y}}_{i}=j$ and 0 otherwise.

Subsequently, the label with the higher frequency of occurrence is identified as follows:

(8) $$v={\mathrm{argmax}}_{j}V\left(j\right),$$

and the number of predictions is then determined as follows:

(9) $$N_{v}=V\left(v\right)$$

The probability β of the majority predicted label is computed as the ratio between the number of times the majority predicted label occurs and the total number of predictions:

(10) $$\beta =\frac{N_{v}}{N_{\mathrm{seg}}}$$

$N_{\mathrm{seg}}$ is the total number of EEG segments used for predictions, and 0<β≤1.

Consequently, the final decision regarding the claimed user’s authentication is made based on the probability β, as follows:

(11) $$\hat{z}=\left\{\begin{array}{cc} \mathrm{genuine}, & \mathrm{if}\;\beta \geq \theta \\ \mathrm{imposter}, & \mathrm{otherwise} \end{array}\right.$$

where threshold θ is a predefined value that determines whether $\hat{z}$ is defined as a genuine user or an imposter.

### 3.5. Validation Procedure

#### 3.5.1. EEG Dataset

In this study, a dataset representing a complete record of P300 evoked potentials during a spelling task was employed, recorded using BCI2000 [63]. The dataset contains data from eight participants and was utilized to assess P300-based BCI performance in individuals with amyotrophic lateral sclerosis (ALS), focusing on one of 36 different characters. EEG signals were recorded from eight channels, placed according to the 10-10 International system (Fz, Cz, Pz, Oz, P3, P4, PO7, and PO8) using active electrodes. The dataset includes information such as stimulus types (target and non-target). The EEG data were digitized at a rate of 256 Hz and band-pass filtered between 1 Hz and 50 Hz. The participants were instructed to spell seven predetermined words of five characters (runs) by controlling a P300 speller matrix. For each character selection (trial), all rows and columns were intensified 10 times (stimuli repetitions), so each item on the interface was intensified 20 times. In the first three runs (15 trials in total), the EEG data were stored to perform a calibration of the BCI classifier without participant feedback, followed by the classifier determining the weights (i.e., classifier coefficients). These weights were later used during the subsequent four testing runs (i.e., runs 4–7) with participant feedback. The dataset was divided into a training set and a testing set. The training set, representing 40% of the data, corresponded to runs 1–3, while the testing set, accounting for the remaining 60%, included runs 4–7.

#### 3.5.2. Data Preprocessing

In the initial phase of the analysis, various signal processing techniques were implemented using custom Python code and the MNE library to obtain a clean EEG signal for further processing. The preprocessing pipeline comprised four main steps: filtering, re-referencing, independent component analysis (ICA) for artifact removal, and windowing of data. The first step in preprocessing involved applying a band-pass filter, which was implemented using a finite impulse response (FIR) with a frequency range of 1–50 Hz, designed using the firwin method. The filter length was set to auto for optimal filtering performance, determined automatically by the MNE library [64]. This filter configuration effectively reduced noise and removed irrelevant frequency components, preserving the signal quality. The EEG signals were re-referenced to the average of all recorded channels using MNE’s average reference method. This ensured that the data rank was preserved, preventing problems in subsequent analyses such as ICA [65]. Including all channels in the re-referencing step helped reduce common noise and enhance the overall signal-to-noise ratio. To complement the filtering, ICA was performed using the fastica algorithm, configured with six components to ensure reproducibility. The algorithm was fitted to the filtered and re-referenced EEG data. Components were inspected manually for artifacts such as eye blinks or muscle movements. This ensured that only artifact-free data were passed to the next stages of analysis. To confirm the efficiency of preprocessing, the EEG data were then visualized before and after preprocessing using ICA to ensure any remaining noise or artifacts were identified. The cleaned data allowed the classifiers to focus on the relevant patterns in the EEG signals. Without proper preprocessing, the model would likely struggle with noisy input, leading to poor performance and unreliable user identification and authentication results.

#### 3.5.3. EEG Data Segmentation

The next component involved generating data segments through windowing. In this case, consecutive segments of 512 samples were extracted from the EEG data, with a 50% overlap applied. This overlapping ensures that important patterns within the EEG segments are not missed during the analysis. Overall, the windowing process allows meaningful patterns to be extracted from the EEG data. This data segmentation ensures that crucial temporal features are preserved, while the overlap helps maintain the continuity of information. If performed improperly, the model could miss important features, negatively impacting the identification and authentication processes.

#### 3.5.4. EEG-Based User Identification Framework

**Figure 3:**
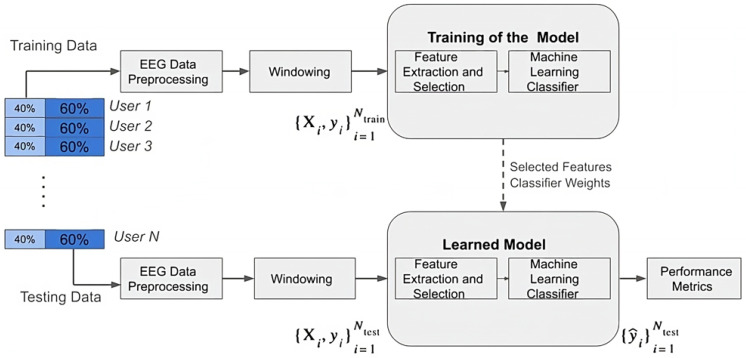
EEG-based user identification framework.

The EEG-based user identification framework for the proposed study is illustrated in Figure 3. The objective of this framework is to validate the identification task described in the above section. To address the identification problem, the dataset is subdivided into two datasets for each user: a training dataset comprising 40% of the complete EEG data and a testing dataset comprising the remaining 60% of the EEG data.

In this framework, the EEG data segments from all users are processed using the proposed classifier. The framework is divided into two phases: a training phase and a testing phase. During the training phase, 40% of the EEG data from all users are utilized. Then, the following steps are performed: first, the data are preprocessed through filtering, referencing, implementation of ICA, and segmentation of data samples using windowing, which generates the output. The training dataset is represented as ${\left\{X_{i},y_{i}\right\}}_{i=1}^{N_{\mathrm{train}}}$, where $N_{\mathrm{train}}$ denotes the number of segments in the training dataset. Before passing these data segments through the machine learning model, relevant features are selected from each segment.

In the testing phase, the remaining 60% of the dataset comprising unseen data is utilized to evaluate the model for the identification process. These data are represented as ${\left\{X_{i},y_{i}\right\}}_{i=1}^{N_{\mathrm{test}}}$, obtained through preprocessing and sequential segmentation of the EEG data. These EEG segments are then passed through the trained model, where the relevant features are extracted using feature selection. These features are finally passed through the machine learning model to predict labels from unseen EEG data ${\left\{{\hat{y}}_{i}\right\}}_{i=1}^{N_{\mathrm{test}}}$. Here, $N_{\mathrm{test}}$ represents the number of segments in the testing dataset. The performance metrics are then computed to analyze the effectiveness of the user identification process.

#### 3.5.5. EEG-Based User Authentication Framework

For the evaluation of the user authentication procedure, the leave-one-participant-out (LOPO) cross-validation technique was employed. This method involves the repetitive training of the model on data from all users except one and validating it on the excluded user. This process ensures that the model is tested on unseen data from each individual user, assessing its ability to distinguish between genuine users and imposters. By repeating this process for each user, the robustness and generalization of the proposed user authentication scheme are assessed across different individuals.

**Figure 4:**
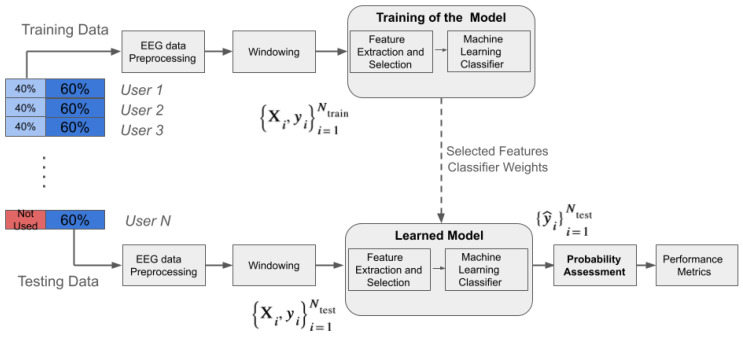
EEG-based user authentication framework.

The proposed EEG-based user authentication framework is shown in Figure 4. This framework is divided into two phases: a training phase and a testing phase. In the training phase, the data from all users except one (this process is repeated for all *N* users) are used. The following steps are performed: preprocessing of the data through filtering, referencing, implementation of ICA, and segmenting the data samples into windows. This process results in an output ${\left\{X_{i},y_{i}\right\}}_{i=1}^{N_{\mathrm{train}}}$, where $N_{\mathrm{train}}$ represents the number of segments in the training dataset.

In the testing phase, the unseen data from all users are used to test the classifier’s performance in the user authentication process. The input feature vector ${\left\{X_{i},y_{i}\right\}}_{i=1}^{N_{\mathrm{test}}}$ is fed into the trained classification model for feature extraction, followed by the prediction of labels ${\left\{{\hat{y}}_{i}\right\}}_{i=1}^{N_{\mathrm{test}}}$ from unseen EEG data segments. These predicted labels are further analyzed through probability assessment to authenticate genuine users and identify imposters based on the predefined threshold values. Testing on unseen data assesses the real-world applicability of the model. High performance in this phase indicates that the model can generalize well, meaning it can identify or authenticate users accurately when presented with new EEG segments.

#### 3.5.6. Performance Metrics

The performance of the user identification and authentication frameworks was evaluated using several key statistical metrics: accuracy, precision, sensitivity (recall), F1 score, and specificity for the multi-class problem. These metrics are expressed as follows:

(12) $$\mathrm{Accuracy}=\frac{1}{K}\sum_{k=1}^{K}\frac{TP_{k}+TN_{k}}{TP_{k}+TN_{k}+FP_{k}+FN_{k}}\times 100\%$$

(13) $$\mathrm{Precision}=\frac{1}{K}\sum_{k=1}^{K}\frac{TP_{k}}{TP_{k}+FP_{k}}\times 100\%$$

(14) $$\mathrm{Sensitivity}\;\left(\mathrm{Recall}\right)=\frac{1}{K}\sum_{k=1}^{K}\frac{TP_{k}}{TP_{k}+FN_{k}}\times 100\%$$

(15) $$F1\;\mathrm{score}=\frac{2\times \mathrm{Recall}\times \mathrm{Precision}}{\mathrm{Recall}+\;\mathrm{Precision}}$$

(16) $$\mathrm{Specificity}=\frac{1}{K}\sum_{k=1}^{K}\frac{TN_{k}}{TN_{k}+FP_{k}}\times 100\%$$

where TP represents true positives, FN represents false negatives, FP represents false positives, *k* is a specific class, and *K* represents the number of all classes. Accuracy is the percentage of correct predictions out of all observations, precision is the average percentage of correctly predicted positive observations out of all predicted positive observations, while sensitivity (recall) is the average percentage of correctly predicted positive observations out of all actual positive observations. The F1 score is the weighted average of precision and recall. Specificity, also known as the true negative rate, measures the proportion of actual negative instances correctly identified out of all true negative instances.

A confusion matrix was also utilized to evaluate the performance of the proposed user identification scheme. The confusion matrix provides a detailed breakdown of the classification results, allowing analysis of the model’s ability to correctly authenticate genuine users and detect impostors. Additionally, the confusion matrix helps visualize the distribution of true positives (TP), true negatives (TN), false positives (FP), and false negatives (FN), providing significant insight into the user authentication technique and its strengths and weaknesses. By comparing the predicted labels with the ground truth, metrics such as accuracy, precision, recall, and specificity were calculated.

The proposed framework was also assessed using error rates such as the false acceptance rate (FAR), i.e., when a user is verified incorrectly as a claimed user, and the false rejection rate (FRR), i.e., when a user is incorrectly denied access despite being the claimed user. The FAR and FRR are calculated as follows:

(17) $$\mathrm{FAR}=\frac{\mathrm{No}.\;\mathrm{of}\;\mathrm{imposters}\;\mathrm{verified}\;\mathrm{as}\;\mathrm{genuine}\;\mathrm{users}}{\mathrm{Total}\;\mathrm{number}\;\mathrm{of}\;\mathrm{attempts}}$$

(18) $$\mathrm{FRR}=\frac{\mathrm{No}.\;\mathrm{of}\;\mathrm{genuine}\;\mathrm{users}\;\mathrm{verified}\;\mathrm{as}\;\mathrm{imposters}}{\mathrm{Total}\;\mathrm{number}\;\mathrm{of}\;\mathrm{attempts}}$$

The FAR scheme is better than the FRR scheme since the FAR determines false identities. The classification accuracy, error rates, ROC curves, DET curves, and other evaluation parameters were statistically calculated. The receiver operating characteristic (ROC) curve is a plot that is utilized to visualize system performance. It is a graphical representation of the comparison between the TPR and the FPR. The positive values on the *x*-axis represent the TPR, and the negative values on the *y*-axis represent the FPR. The detection error trade-off (DET) is a graphical representation of the trade-off between the false positive rate (FPR) and the false negative rate (FNR). The curve demonstrates the relationship between these two error rates. The FPR is represented on the *x*-axis and the FNR on the *y*-axis. The probability threshold ensures that predictions are not binary but probabilistic, leading to better user identification and authentication. It directly impacts the FAR and FRR, determining an optimal threshold that helps balance the trade-off between falsely accepting imposters (FAR) and falsely rejecting genuine users (FRR).

#### 3.5.7. Statistical Analysis

To assess the empirical chance level, the target labels in the training data were shuffled multiple times to break any potential relationship between features and labels. The model was then trained on these shuffled labels and evaluated on the original test set. This process was repeated 100 times to generate an empirical distribution of the model’s performance under random label shuffling. The mean and standard deviation of this distribution provided insights into the classifier’s performance relative to the empirical chance level. By training on shuffled labels and comparing the results to those obtained from the actual data, the classifier’s performance was assessed against this empirical chance baseline. This procedure ensures that the model’s output is based on real patterns rather than random associations, verifying that its performance improves significantly beyond chance.

## 4. Results and Analysis

This section briefly presents and demonstrates the procedure used to evaluate the proposed method and investigates the impact of using EEG signals for its implementation. The scheme’s performance for the user identification and authentication tasks is presented, and a detailed comparison with related research is also discussed.

### 4.1. Classifier Implementation

All the experiments in this section were implemented using Python (version 3.8). EEG signals associated with the P300 speller were utilized, and some studies have demonstrated that these data can lead to higher accuracy in EEG-based user authentication [66,67]. The experimental setup and data processing were carried out within Jupyter Notebooks. All the results were analyzed and performed in the local Jupyter Notebook environment.

An EEG-based user authentication system depends on the number of EEG channels used and the length of the signal. Stand-alone classifiers, along with the proposed classifier, were trained and tested on both the training and testing datasets for benchmarking. The best accuracy results were computed during the training and testing phases using EEG data from all users. The best performance for the user identification and authentication tasks was reported using various evaluation metrics. The parameters and hyperparameters employed for these multi-class classifiers are presented in Table 2.

The values of the proposed classifier’s parameters and hyperparameters are shown in Table 3. The different metrics, such as precision, recall, F1 score, and accuracy, used for the multi-class classifiers are presented in Table 4.

### 4.2. User Identification Task Results

**Figure 5:**
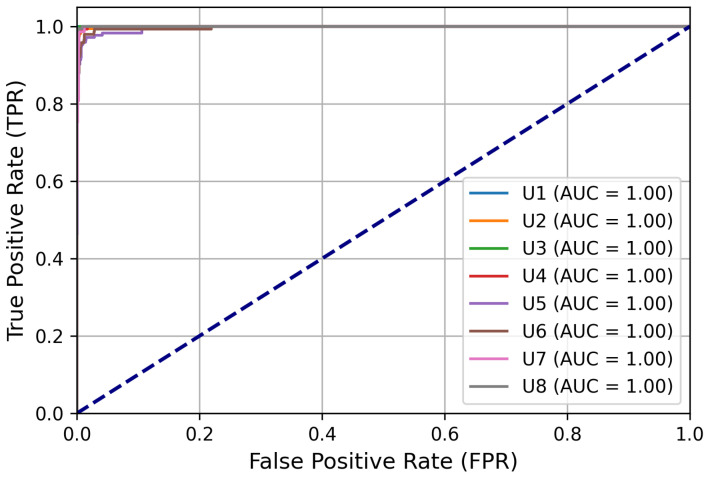
ROC curves illustrating classification performance for EEG-based user identification. The dotted line represents the baseline for random classification. It is a diagonal line with a slope of 1, running from the bottom-left corner (0,0) to the top-right corner (1,1).

The FFNN was trained with EEG data segments following the procedure outlined in the user identification task section. The final evaluation was conducted within the user identification framework phase. EEG data segments in the training dataset were used as input, with feature vectors extracted using mutual information-based feature selection criteria. The effectiveness of the classifier was assessed using the AUC-ROC metric to distinguish between different users based on their brain patterns. Given that there were multiple classes, each representing a different user, the multi-class AUC-ROC curves provided a broad view of the model’s ability to identify users accurately. The AUC-ROC plot in Figure 5 shows significant outcomes, with a high true positive rate (TPR) for all users, ensuring accurate identification.

**Figure 6:**
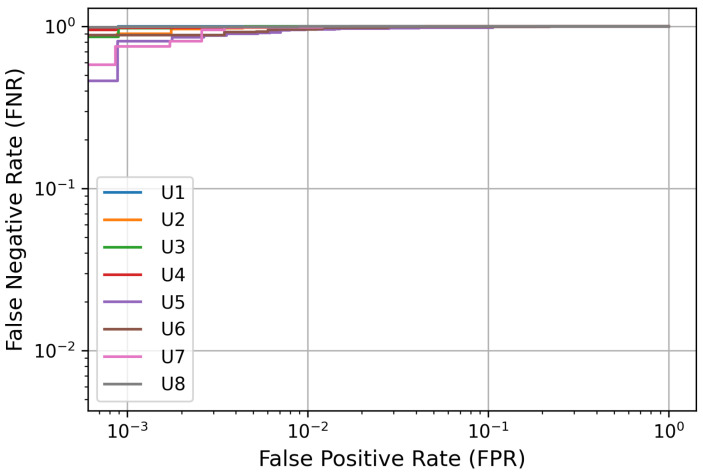
Detection error trade-off (DET) curves for EEG-based user identification.

A detection error trade-off (DET) curve was generated to further evaluate the performance of the EEG-based user identification scheme. Figure 6 illustrates the DET curves for all users, showcasing how errors can be traded off against each user. The classification model output demonstrates significant performance across multiple users, achieving an area under the curve (AUC) value of 1 for each user. The change in the curve’s shape suggests a strong balance between false positive and false negative rates, indicating the model’s perfect discrimination potential in effectively distinguishing between different user classes.

**Figure 7:**
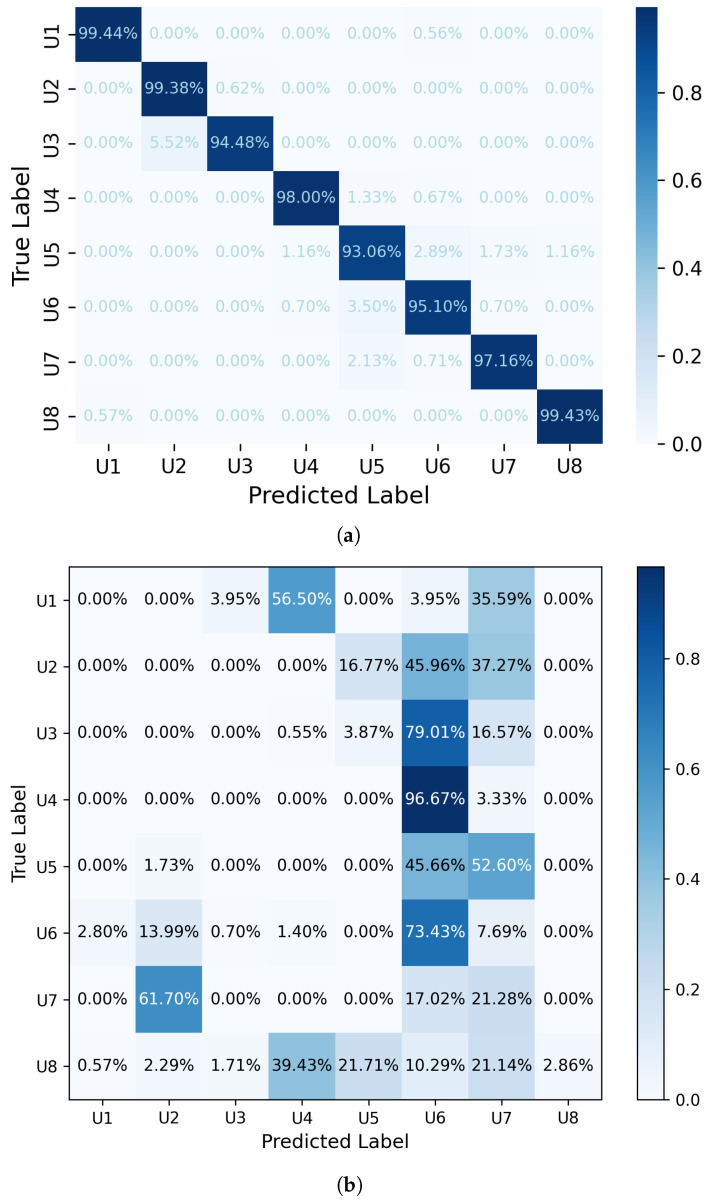
(**a**) Confusion matrix showing the model’s classification accuracy for user identification. (**b**) Confusion matrix after label shuffling over *n* iterations for user identification.

Table 5 demonstrates the average accuracy of users using multiple classifiers and the proposed model for the recognition scheme. To provide a detailed assessment and assess model performance for individual users, accuracy was evaluated separately during the testing phase. Figure 7a visually represents the model’s performance, showing the distribution of correct and incorrect predictions across different classes.

This figure illustrates the accuracy of each user separately. While Users U3 and U5 exhibit slightly lower accuracies compared to the others, the overall model accuracy is 97%, indicating strong performance in identifying users from EEG signals. After shuffling the labels across *n* iterations, a confusion matrix was created to assess the model’s robustness. In order to evaluate the model’s performance against random guessing, this matrix was used as a benchmark. This procedure helps determine the extent to which the model relies on real EEG patterns as opposed to random correlations by comparing the outcomes of the original model with those obtained from shuffled labels. After label shuffling, the confusion matrix shown in Figure 7b displays much lower accuracy rates, indicating that the model’s successful recognition was not the result of random chance. This study demonstrates the model’s potential for real-time applications in user authentication by confirming its effectiveness in distinguishing people based on genuine EEG data.

### 4.3. User Authentication Task Results

The user authentication framework employs the same procedure and scheme as the identification framework. The main difference lies in utilizing predicted labels to classify users, followed by a probability assessment to determine the responses as either “valid” or “invalid”. The proposed model demonstrates a significantly improved accuracy, especially with the proposed classifier in EEG-based user authentication. To test the model’s adaptability in identifying new EEG patterns, it was evaluated on a separate dataset (i.e., the testing dataset) that was not used during training. This approach enhanced the likelihood that the model could generalize and recognize patterns from different users beyond the training data.

**Figure 8:**
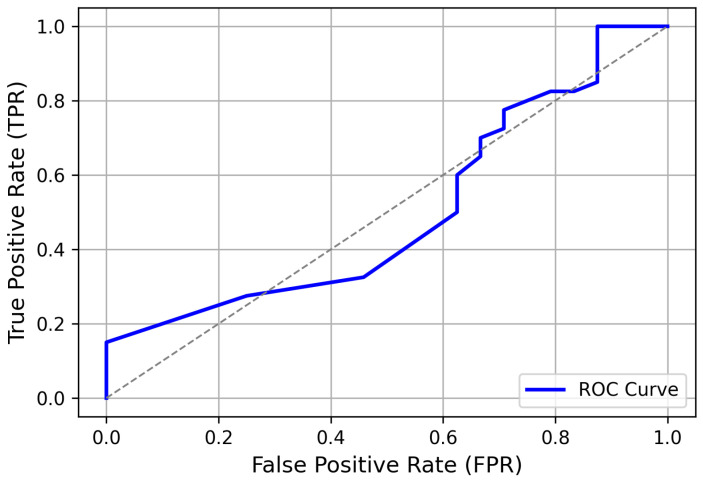
ROC curve depicting the model’s performance in EEG-based user authentication. The dotted line represents the chance level or random guessing.

This dataset was then employed to assess the model’s performance in authenticating new users. By exposing the model to **“unseen”** data from the testing dataset, it effectively generalized to authenticate new users based on their EEG records. The ROC plot in Figure 8 illustrates significant outcomes, with a high true positive rate (TPR), ensuring accurate authentication of genuine users while effectively minimizing the false positive rate (FPR) to detect impostors. The proposed model achieved a significant increase in the TPR in recognizing genuine users based on a predefined threshold value, achieving 97.02% accuracy in EEG-based user authentication. These results are noteworthy and distinguishable from previous research, which did not include such a classification procedure for user authentication.

**Figure 9:**
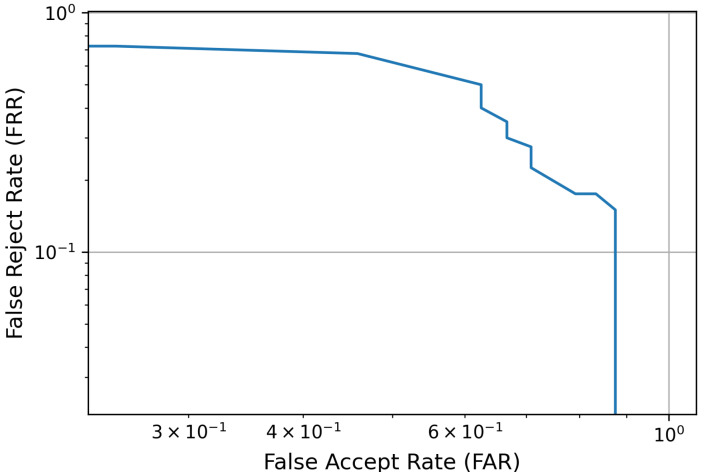
Detection error trade-off (DET) curve showcasing model performance in EEG-based user authentication. The grey lines represent grid lines to aid in the interpretation of the DET curve values, especially given the logarithmic scales used for both axes.

Subsequently, based on selected features using mutual information (MI), scores for genuine and impostor cases were generated, with label 1 indicating a genuine score (correct user) and label 0 indicating an impostor score (incorrect user). The threshold directly influenced the performance of the user authentication model: a lower value increased the false rejection rate (FRR), while a higher value increased the false acceptance rate (FAR). Using the proposed model, a detection error trade-off (DET) plot was generated to assess the performance of EEG-based user authentication, as depicted in Figure 9.

**Figure 10:**
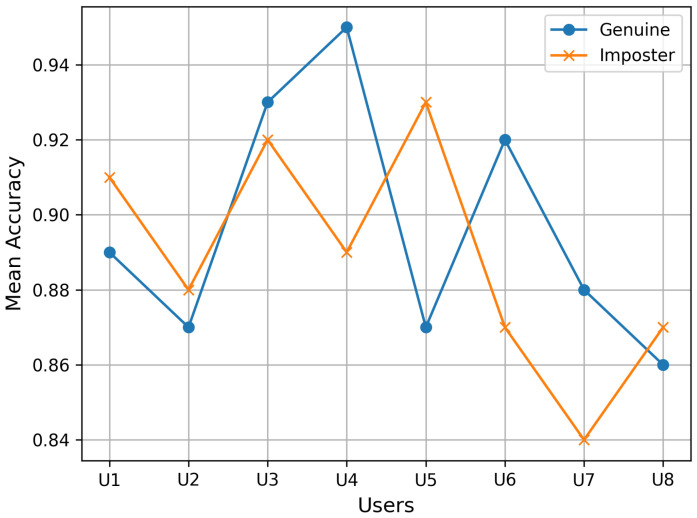
Authentication accuracy for genuine users vs. imposters in EEG-based authentication.

Additionally, the overall accuracy of EEG-based user authentication was calculated by taking the ratio of the number of true positive classifications (genuine users correctly identified) to the total number of classifications. This accuracy metric illustrated the model’s capability to accept genuine users while minimizing false positives, as shown in Figure 10.

Statistical analyses were carried out to further demonstrate the effectiveness of the proposed approach. For randomly distributed data, a Wilcoxon signed-rank test was used to evaluate the accuracy, false rejection rate (FRR), and false acceptance rate (FAR). ANOVA and Tukey’s HSD post-hoc analysis illustrated that the classifiers’ differences in authentication accuracy were statistically significant (*p*-values < 0.05). The *p*-values for each user varied from 0.0001 to 0.0007, suggesting that it was highly unlikely that the observed performance differences were the result of chance. These results indicate that the classifiers’ performance changed, with the FFNN achieving the best accuracy across users (with a range from 0.96 to 0.99). The significant differences between the FFNN and other classifiers (KNN, SVM, RF, and XGB) emphasize its effectiveness in user authentication tasks.

## 5. Discussion and Comparative Analysis with Related Studies

The proposed feedforward neural network (FFNN) significantly improves EEG-based user identification and authentication, demonstrating a marked enhancement in accuracy compared to previous studies. Specifically, when compared to the study by Waili et al. [47], which used a more limited dataset (six users and 19 channels) and employed wavelet decomposition on alpha and beta bands, the proposed method shows a substantial improvement in user recognition accuracy. While Waili et al. achieved 73% accuracy using multi-layer perceptron (MLP) neural networks, the FFNN in this study achieved 97% accuracy, which is a notable advancement. Table 6 provides an overview of current EEG-based user authentication techniques that utilize neural networks across various datasets, along with a comparative analysis highlighting the advancements and distinctions of the proposed method.

One of the key differences between the proposed approach and prior research lies in the number of EEG channels used. As noted, the use of a single channel for EEG-based authentication, such as in [52], inherently limits accuracy due to the reduced feature complexity across users and tasks. In contrast, our approach utilizes a reduced channel configuration of eight channels, which strikes a balance between practical feasibility and high performance. This approach enhances generalizability to unseen data, which is crucial for real-time applications where computational efficiency is important. The scalability of the system, while still leveraging the minimal channel setup, significantly reinforces security protocols, making it more suitable for real-time applications compared to studies that rely on larger numbers of channels, such as the 64 channels used by Liew et al. [51].

The comparison with studies like Liew et al. [51], which used 64 channels and advanced feature extraction techniques like cross-correlation and coherence, highlights a trade-off between accuracy and practicality. While their method achieved 87% accuracy, it is less feasible for real-time applications, where a large number of channels may not be practical due to data privacy concerns and computational requirements. The proposed study circumvents these challenges by focusing on optimizing feature selection using mutual information analysis and selected features, particularly in the power spectral density (PSD) domain, to enhance feature discriminability without relying on a vast array of channels.

Moreover, Bhateja et al. [52] highlighted the limitations of using signal fusion in single-channel EEG mixed with Electrooculography (EOG) signals, which could impose further constraints on authentication accuracy. In contrast, the proposed method emphasizes feature extraction and selection over signal fusion, which not only improves accuracy but also addresses privacy concerns by minimizing the direct processing of raw EEG data. By reducing the number of channels and focusing on relevant features, the method provides a more efficient, practical, and privacy-preserving solution for EEG-based user authentication.

Despite the promising results, several limitations and factors must be considered when interpreting these findings. First, the dataset used in this study, while consisting of eight users and eight channels, may not fully represent the variability seen in larger, more diverse populations. The small user pool limits the study’s ability to generalize to a broader population, especially in more heterogeneous environments or in real-world scenarios. Additionally, the reliance on a specific set of features, such as those derived from PSD, may not capture all relevant aspects of the EEG signal, particularly in more complex tasks or individuals with significantly different brainwave patterns.

Furthermore, model assumptions may not apply to other datasets or tasks, such as the suitability of FFNNs for feature extraction. Models like EEGNet, which is designed for EEG analysis and efficiently captures spatial and temporal data using depthwise and separable convolutions, could be used in future research. Performance may be further improved by hybrid strategies that combine EEGNet with conventional feature extraction. In addition, by extending methods or adding domain-specific knowledge, biases in feature selection from mutual information analysis could be minimized. Evaluating models such as EEGNet on a variety of tasks could enhance their classification accuracy and robustness.

In summary, the proposed EEG-based user authentication system demonstrates improved performance compared to existing methods by optimizing the trade-off between accuracy, efficiency, and practicality. While the approach offers several advantages in terms of scalability, real-time application suitability, and privacy preservation, further exploration of model assumptions, dataset constraints, and feature selection biases is necessary to improve generalizability and performance. Continued development in these areas could lead to even more reliable and secure EEG-based authentication systems, paving the way for real-world deployment in high-security applications.

## 6. Conclusions and Future Work

This study addresses the practical challenges in EEG-based user authentication, such as comprehensiveness and privacy. To achieve this objective, an EEG-based user identification and authentication scheme is proposed that utilizes a feedforward neural network (FFNN) to extract relevant features from EEG signals. The system then verifies the user’s claimed identity by comparing the similarities between the stored data and the selected EEG features. Privacy preservation is ensured through multiple mechanisms: (1) only the extracted features, not the raw EEG data, are stored, thus preventing any reconstruction of the original EEG signals; (2) all stored data are obscured, preventing the detection of any specific individual; and (3) robust encryption techniques are applied to secure both the stored features and transmitted data, preventing unauthorized access. Furthermore, the proposed method eliminates the possibility of users accessing their own EEG data, thereby further safeguarding privacy. Another advantage of this approach is its comprehensiveness, as it can be used for all users, including those who are not part of the training database. This method demonstrates ease of use and can operate with a smaller number of channels. Consequently, the results show an accuracy of 97%, which is superior to that of other related studies.

While this research demonstrates the potential of EEG-based user authentication, addressing data privacy and model robustness remains essential. Robustness can be evaluated through adversarial attack simulations to assess the system’s resilience against malicious attempts. Enhancing feature extraction methods may improve the system’s ability to capture subtle user-specific patterns, while more sophisticated machine learning architectures can better exploit the spatial and temporal dependencies in EEG data. Exploring transfer learning, real-time implementation, and integrating user feedback and interaction are critical steps toward practical deployment. Furthermore, evaluating the system across diverse datasets and exploring its potential for secure access to data centers, where critical data and servers are kept, as well as bank vaults, will enhance its broader applicability and impact. These advancements will contribute to the development of secure, reliable, and broadly applicable EEG-based user authentication systems.

## Figures and Tables

**Table 1:** Comprehensive overview of reviewed studies on EEG-based user authentication.

Research	Year	Channels	Protocol	Users	Feature Extraction	Classifiers	Accuracy
Wailiaiprasitporn [39]	2020	32	Audio	32	NM	SVM	88%
K.Bialas et al. [40]	2022	32	Hybrid	N/A	AR	Fast Forest	83%
Leon et al. [41]	2022	16	VEP	12	CFS	SVM	72%
Schons et al. [42]	2018	64	Rest	109	NM	CNN	99%
Bingkun et al. [43]	2022	16	Motor	9	PSD	CNN	93%
Debie et al. [44]	2021	64	ERP	54	PSD	CNN	99%
Mariorana et al. [45]	2017	19	Rest	45	AR, MFCC	HMM	98%
Kim et al. [46]	2019	16	Rest	109	NM	FM	77%
Waili et al. [47]	2019	19	Rest	6	Wavelets	MLP	73%
Haukipuro et al. [49]	2018	1	Image/Motor	27	PSD, MFCC	MLP	83%
N. Alzahab et al. [50]	2022	4	Audio	N/M	CFS	MLP, XBoost, SVM	69%
Liew et al. [51]	2018	64	VEP	37	WPD	KNN, FRNN	87%
Bhateja et al. [52]	2019	1	Blinking	15	Wavelet, ICA	ANN	80%

**Table 2:** Summary of the parameters and hyperparameters utilized for the various classifiers in the analysis.

Model	Parameters/Hyperparameters
Support Vector Machine (SVM)	Kernel: Linear
	C (Regularization Parameter): 1
K-Nearest Neighbors (KNN)	Number of Neighbors: 3
Random Forest (RF)	Number of Trees: 100
	Random State: 123
XGBoost (XGB)	Number of Boosting Rounds: 100
	Max Depth (Step-Size Shrinkage): 0.1

**Table 3:** Details of the parameters and hyperparameters used for the proposed feedforward neural network (FFNN) model.

Parameter	Value
Learning Rate	0.001
Loss Function	Cross-entropy
Number of Epochs	50
Batch Size	32
Task Duration	60 s
Input Size	10
Optimizer	Adam optimizer
Sampling Frequency	256 Hz
Number of Classes	8
Window Size	2 s
Stride	256
Number of Samples	1357
Window Overlapping	1 s

**Table 4:** Performance metrics used to assess the efficacy of the classifiers.

Model	Precision	Recall	F1 Score	Accuracy
SVM	0.84	0.84	0.84	84.08%
KNN	0.82	0.82	0.82	82.28%
RF	0.84	0.84	0.84	84.14%
XGB	0.84	0.84	0.84	83.71%
FFNN	0.97	0.97	0.97	97.02%

**Table 5:** Comparison of average authentication accuracy and statistical significance across users for various classifiers and the proposed FFNN model.

Users	SVM	KNN	RF	XGB	FFNN	*p*-Value
U1	0.83	0.81	0.86	0.84	0.98	0.0001
U2	0.85	0.83	0.86	0.82	0.97	0.0003
U3	0.87	0.85	0.85	0.83	0.98	0.0002
U4	0.83	0.80	0.86	0.83	0.97	0.0005
U5	0.84	0.79	0.84	0.82	0.96	0.0004
U6	0.83	0.83	0.85	0.83	0.98	0.0006
U7	0.84	0.82	0.85	0.84	0.97	0.0007
U8	0.87	0.85	0.80	0.87	0.99	0.0002

**Table 6:** Comparative analysis of EEG-based user authentication with existing studies.

Paper	Users	Layers	Channels	Accuracy	Classifiers
Waili et al. [47]	6	3	19	73%	MLP
Haukipuro et al. [49]	27	3	1	83%	MLP
N. Alzahab et al. [50]	N/M	5	4	69%	MLP, SVM, XGBoost
Liew et al. [51]	15	3	64	87%	KNN, FRNN
Bhateja et al. [52]	15	—	1	80%	ANN
Proposed Work	8	3	8	97%	FFNN

## Data Availability

A publicly available dataset was analyzed in this study, which is available at https://bnci-horizon-2020.eu/database/data-sets (accessed on 15 February 2024).

## Abbreviations

The following abbreviations are used in this manuscript:

VEP	Visual Evoked Potential
PINs	Personal Identification Numbers
ERP	Event-Related Potential
HMM	Hidden Markov Model
FRNN	Fuzzy Recurrent Neural Network
MFCC	Mel-Frequency Cepstral Coefficient
NM	Neural Model
FM	Factorization Machine
CFS	Continuous Flash Suppression
AR	Auto Regression
ANN	Artificial Neural Network
WPD	Wavelet Packet Decomposition
